# Untangling the Evolution of the Receptor-Binding Motif of SARS-CoV-2

**DOI:** 10.1007/s00239-024-10175-y

**Published:** 2024-05-22

**Authors:** Luis Delaye, Lizbeth Román-Padilla

**Affiliations:** Departamento de Ingeniería Genética, Cinvestav Unidad Irapuato, Km 9.6 Libramiento Norte Carretera Irapuato-León, C.P. 36824 Irapuato, Gto. Mexico

**Keywords:** Recombination, Viral evolution, Bayes factors, Dissonance, Information content

## Abstract

The spike protein determines the host-range specificity of coronaviruses. In particular, the Receptor-Binding Motif in the spike protein from SARS-CoV-2 contains the amino acids involved in molecular recognition of the host Angiotensin Converting Enzyme 2. Therefore, to understand how SARS-CoV-2 acquired its capacity to infect humans it is necessary to reconstruct the evolution of this important motif. Early during the pandemic, it was proposed that the SARS-CoV-2 Receptor-Binding Domain was acquired via recombination with a pangolin infecting coronavirus. This proposal was challenged by an alternative explanation that suggested that the Receptor-Binding Domain from SARS-CoV-2 did not originated via recombination with a coronavirus from a pangolin. Instead, this alternative hypothesis proposed that the Receptor-Binding Motif from the bat coronavirus RaTG13, was acquired via recombination with an unidentified coronavirus. And as a consequence of this event, the Receptor-Binding Domain from the pangolin coronavirus appeared as phylogenetically closer to SARS-CoV-2. Recently, the genomes from coronaviruses from Cambodia (bat_RShST182/200) and Laos (BANAL-20-52/103/247) which are closely related to SARS-CoV-2 were reported. However, no detailed analysis of the evolution of the Receptor-Binding Motif from these coronaviruses was reported. Here we revisit the evolution of the Receptor-Binding Domain and Motif in the light of the novel coronavirus genome sequences. Specifically, we wanted to test whether the above coronaviruses from Cambodia and Laos were the source of the Receptor-Binding Domain from RaTG13. We found that the Receptor-Binding Motif from these coronaviruses is phylogenetically closer to SARS-CoV-2 than to RaTG13. Therefore, the source of the Receptor-Binding Domain from RaTG13 is still unidentified. In accordance with previous studies, our results are consistent with the hypothesis that the Receptor-Binding Motif from SARS-CoV-2 evolved by vertical inheritance from a bat-infecting population of coronaviruses.

**Supplementary Information:**

The online version contains supplementary material available at 10.1007/s00239-024-10175-y.

## Introduction

How the coronavirus SARS-CoV-2 evolved to infect humans continues to be an active area of research. To understand the origin of the zoonosis it is crucial to identify the closest viral wild population from which SARS-CoV-2 originated. Very early during the pandemic, the bat coronavirus RaTG13 from China’s Yunnan province, was identified as the most closely related to SARS-CoV-2, showing an average genome-wide nucleotide identity of 96.1% (Zhou et al. 2020).

This was followed by the proposal that the Receptor-Binding Domain (RBD) of the spike protein from SARS-CoV-2 was acquired by recombination with pangolin-infecting coronaviruses (Li et al. 2020; Zhang et al. 2020; Lam et al. 2020). These coronaviruses were identified in two provinces of China: Guangxi (GX) and Guangdong (GD). This proposal was based on the high similarity of the RBD from the pangolin-infecting coronaviruses to that from SARS-CoV-2.

The origin of the RBD from SARS-CoV-2 by recombination was challenged by Boni et al. (2020). According to Boni et al. (2020) the Receptor-Binding Motif (RBM) has a different phylogenetic history than the RBD in RaTG13. The Receptor-Binding Motif (RBM) is a hypervariable loop located within the RBD that contains the amino acids that recognize the Angiotensin-converting enzyme 2 (ACE2) host protein in SARS-CoV-2. And it is known that strains of SARS-CoV isolated from different hosts show varying affinities for human ACE2 (reviewed in Cui et al. 2019). According to Boni et al. (2020) it was the RBM from RaTG13 the one that evolved by recombination with an unknown coronavirus; and as a result of this recombination, the RBM from the pangolin-infecting coronavirus appeared as phylogenetically closer to SARS-CoV-2 than that of RaTG13. Following Boni et al. (2020) a lineage of sarbecovirus capable of infecting humans circulated in bats for decades before the pandemic.

Recently, two research groups reported the genome sequences of coronaviruses isolated from bats that are closely related to SARS-CoV-2. These are two coronaviruses isolated from Cambodia (bat_RShSTT182 and bat_RShSTT200) that show a genome-wide nucleotide identity of 92.6% to SARS-CoV-2 (Delaune et al. 2021); and five coronaviruses isolated from Laos (BANAL-20-52, BANAL-20-103, BANAL-20-116, BANAL-20-236 and BANAL-20-247) that show levels of average genome-wide nucleotide identity that range from 96.8 to 97.4% (Temmam et al. 2022). These last coronaviruses from Cambodia are on average more similar to SARS-CoV-2 than RaTG13.

Phylogenetic analyses of these coronaviruses from Cambodia and Laos showed that recombination played an important role in their evolution, as is common for other coronaviruses (Delaune et al. 2021; Lytras et al. 2022; Temmam et al. 2022). Nevertheless, previous studies did not address specifically the evolution of the RBM in these novel coronavirus sequences. In particular, Boni et al. (2020) showed that the RBD from SARS-CoV-2 did not originate by recombining with a pangolin-infecting coronavirus. They also showed that the RBM from RaTG13 was acquired via recombination with an unidentified coronavirus. The objective of this work is to test (with more in deep phylogenetic and recombination analyses) whether the coronaviruses from Cambodia (bat_RShSTT182 and bat_RShSTT200) and/or Laos (BANAL-20-52/103/247) were the source of the RBM from RaTG13; and to infer the recent phylogenetic history of the RBD and RBM from SARS-CoV-2 in the context of the novel genome sequences.

## Results

We first selected the genes coding for the spike protein from a set of coronaviruses most closely related to SARS-CoV-2 (Table 1). These were identified following the phylogenetic studies from the following: (Li et al. 2020; Zhang et al. 2020; Boni et al. 2020; Delaune et al. 2021; Lytras et al. 2022; Temmam et al. 2022). More specifically, these coronaviruses were selected because they constitute the nCov clade as defined by Lytras et al. (2022) in its recombination-minimized phylogeny. In agreement with the study from Lytras et al. (2022), these coronaviruses tend to form a monophyletic clade along the different recombining segments detected by Delaune et al. (2021). Therefore, the phylogenetic signal among these sequences must inform us mostly about vertical inheritance. The rationale is that having these sequences in the background will make it easier to detect recombination events in the RBM.

**Table 1:** The gene coding for the spike protein from these coronaviruses were considered for this study. All these coronaviruses are closely related to SARS-CoV-2 (Wuhan-Hu-1/2019)

Coronavirus	Accession ID	Host
Wuhan-Hu-1/2019	MN908947.3	Human
BANAL-20-103	MZ937001.1	*Rhinolophus pusillus*
BANAL-20-52	MZ937000.1	*Rhinolophus malayanus*
BANAL-20-247	MZ937004.1	*Rhinolophus malayanus*
RmYN02	EPI_ISL_412977	*Rhinolophus malayanus*
bat_RShSTT182	EPI_ISL_852604	*Rhinolophus shameli*
RaTG13	MN996532.2	*Rhinolophus affinis*
bat_SL_CoVZC45	MG772933.1	*Rhinolophus pusillus*
RsYN04	MZ081380.1	*Rhinolophus stheno*
RacCS203	MW251308.1	*Rhinolophus acuminatus*
Rc_o319	LC556375.1	*Rhinolophus cornutus*
PrC31	MW703458.1	*Rhinolophus* sp.
Guangdong 1	EPI_ISL_410721	*Manis javanica*
Guangxi_P4L	EPI_ISL_410538	*Manis javanica*

**Fig. 1:**
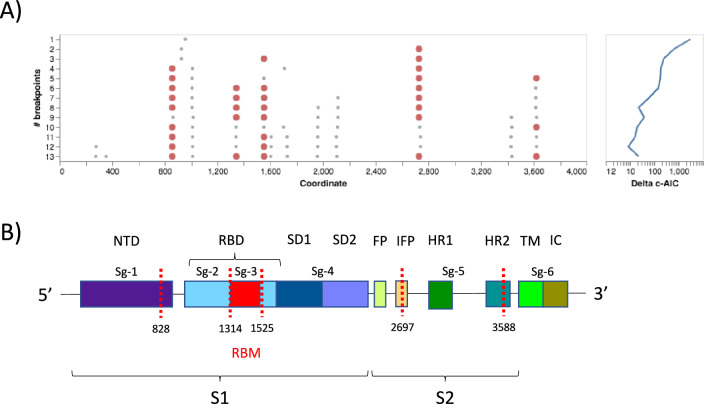
Recombination analysis with GARD of the gene coding for the spike protein from 14 different coronaviruses. 1 **A** Left, orange dots show the best place of inferred breakpoints for each number of breakpoints considered along the multiple sequence alignment. Right, the improvement in the c-AIC score between successive breakpoint numbers (log scale). **B** Location of the six inferred breakpoints (vertical dotted red lines) along the gene sequence of Wuhan-Hu-1/2019. Domain and subdomain structure of the coded protein from Wuhan-Hu-1/2019 is shown as follow: NTD, N-terminal domain; RBD, recognition-binding domain; RBM, recognition-binding motif; SD1 and SD2, subdomains 1 and 2; FP, fusion peptide; HR1 and HR2, heptad repeat 1 and 2; TM, transmembrane region; IC, intracellular domain; S1 and S2, subunits 1 and 2. Sg-1 to Sg-6 stands for segments 1 to 6 as defined by GARD

We next codon-aligned the genes of the spike protein of the selected coronaviruses and searched for recombination breakpoints with the Genetic Algorithm for Recombination Detection (GARD). Briefly, GARD identifies recombination events by finding segment-specific phylogenies along a multiple sequence alignment (Kosakovsky Pond et al. 2006). Most likely recombination breakpoints are depicted by orange dots in Fig. 1A.

**Fig. 2:**
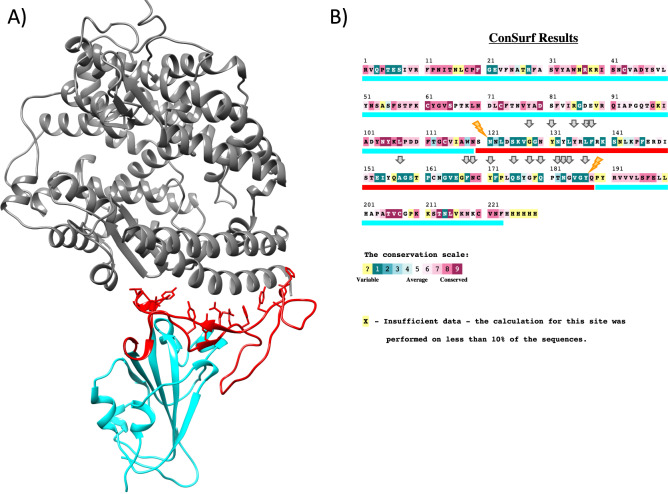
The RBM as defined by Lan et al. (2020) coincides almost perfectly with the hypervariable region as identified by ConSurf and the recombination segment by GARD. **A** Crystal structure of SARS-CoV-2 spike receptor-binding domain bound with ACE2 (PDB: 6M0J). The RBM as defined by Lan et al. (2020) is in color red (residues 438 to 506), ACE2 is shown in gray and the RBD in cyan; **B** Conservation of residues in the RBD according to the ConSurf analysis. The RBM is shown with the red underline and the rest of the RBD in cyan, residues involved in recognition of ACE2 are indicated with gray arrows (Temmam et al. 2022) and recombination sites as inferred by GARD are indicated with yellow rays

**Fig. 3:**
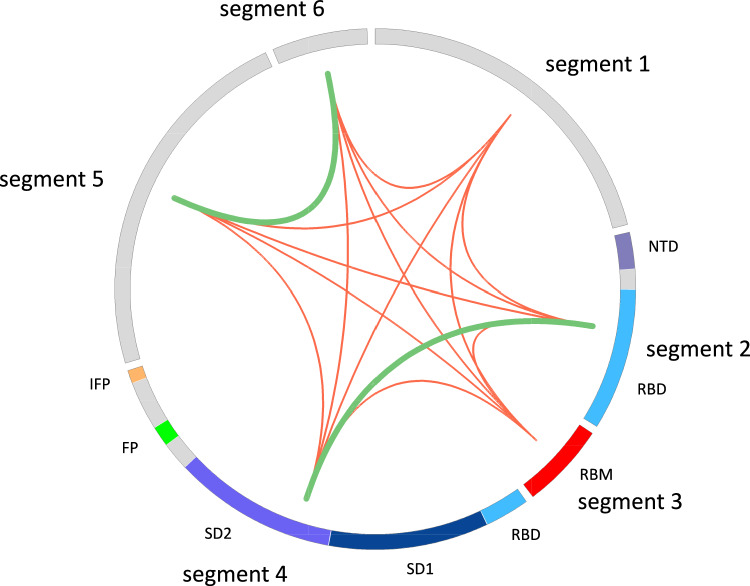
Bayes factor analysis indicates that segments joined by green lines are better described by a single phylogeny and should be concatenated while segments joined by red lines are better described by two different phylogenies. The subdomains and motifs of the spike protein are shown for segments 2, 3 and 4

**Fig. 4:**
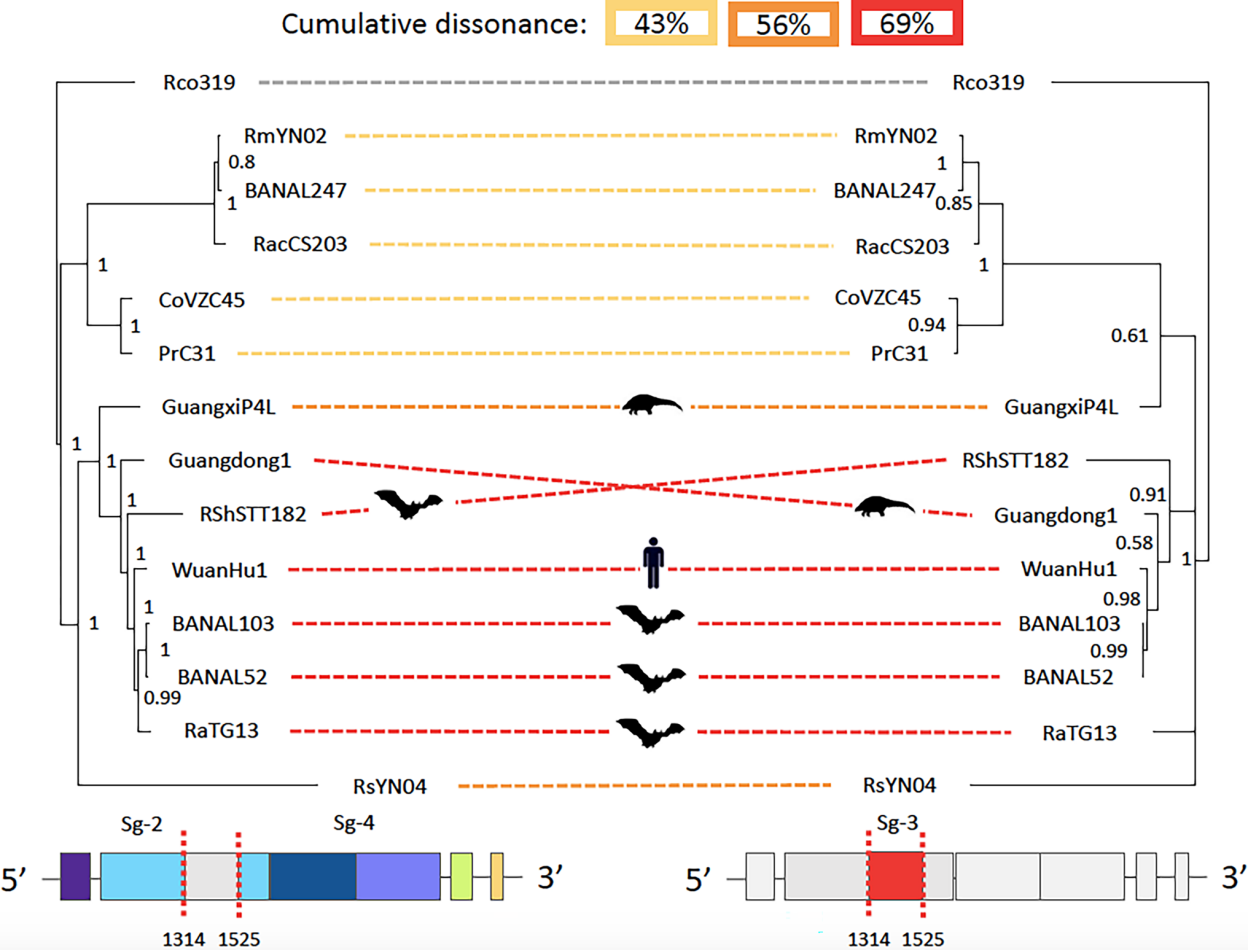
The RBM from RaTG13 has a distinct phylogenetic history than its RBD. Tree on the left is from the concatenation of segments 2 and 4 (containing the RBD); and the tree on the right is from segment 3 (containing the RBM). At the bottom we show segments 2 and 4 (left) and 3 (right) colored by subdomains and motifs. The vertical red lines indicate the recombination breakpoints identified by GARD. At the top of the Figure we show the cumulative dissonance by clades. The hosts are shown for clarity. Note that RaTG13 branches in different clades between the two trees

Accordingly, there are 6 segments most likely free of recombination (Table 2). These five recombination breakpoints map to different domains of the coded protein (Fig. 1B). Note that the RBD is color coded in cyan and the RBM in red (these colors will be maintained through the Figs. 1, 2, 3, 4).

**Table 2:** GARD analysis on the 14 genes coding for the spike protein from selected coronaviruses suggest there are six segments free of recombination

Segment No	Coordinate in alignment	Coordinate in WuhanHu-1/2019	Size of the segment (nuc)	Sequence**
segment 1	1–857	1–827	827	TC**TA**TT
segment 2	858–1343	828–1313	486	TT**CT**AA
segment 3*	1344–1554	1314–1524	211	TA**CA**GA
segment 4	1555–2729	1525–2696	1172	TG**CT**AT
segment 5	2730–3621	2697–3588	892	TC**TC**TC
segment 6	3622–3855	3589–3822	234	ACATA**A**

*This segment coincides with the RBM as defined by Lan et al. (2020)

**The last and first nucleotide of each segment is shown in bold and underlined for the Wuhan-Hu-1/2019 gene sequence

Surprisingly, two of the recombination breakpoints coincide with the limits of the RBM as defined by Lan et al. (2020) and with the hypervariable region as revealed by ConSurf analysis (Fig. 2). ConSurf provides pre-calculated conservation profiles of residues in proteins/structures obtained by using multiple sequence alignments and phylogenetic methods (Ashkenazy et al. 2010; Ben Chorin et al. 2020).

We further evaluated the veracity of recombination breakpoints detected by GARD by using Bayes factors (BF). In brief, for all pairs of segments free of recombination detected by GARD the hypotheses CONCATENATED and SEPARATED were evaluated. The CONCATENATED hypothesis assumes that the evolution of a given pair of segments is better described by a single phylogeny; while the SEPARATED hypothesis assumes that the evolution of a given pair of segments is better described by two independent phylogenies. To evaluate the CONCATENATED hypothesis, the sequences from different segments are concatenated; and in the SEPARATE hypothesis each segment is analyzed separately. The marginal likelihoods are calculated for each hypothesis by the stepping-stone algorithm in MrBayes and then compared. The hypothesis with the largest marginal likelihood is preferred as the one that best-fits the data.

The analysis with Bayes factors showed that the 2nd and 4th segments should be concatenated because their evolution is better described by a single phylogeny; and the same applies for the 5th and 6th segments (Fig. 3). In both cases, the Bayes factor (B_CS_) is larger than 100 which is interpreted as decisive evidence against the SEPARATE hypothesis (Kass and Raftery 1995). Therefore, the evolution of the gene coding for the spike protein for these 14 coronaviruses is better described by 4 recombination breakpoints.

Next, we focused on the phylogenetic history of the concatenated segments 2 and 4 versus the segment 3. Segments 2 and 4 contain a small fraction of the NTD, the RBD (minus the RBM), SD1, SD2, FP and a fragment of the IFP motif (Fig. 3); while segment 3 corresponds to the RBM. The topology of the phylogenetic trees of these segments are almost identical except for three exceptions (Fig. 4). In the first place, the coronavirus Guangxi_P4L is in a different bipartition in the tree inferred from segment 3 (the tree in the right of Fig. 4), although the posterior probability of the internal node supporting this bipartition is low (0.61), raising doubts on its veracity. Second, the coronaviruses Guangdong 1 and bat_RShSTT182 interchange positions between the two trees, but again, the posterior probability supporting the position of Guangdong 1 is low (0.58) in the tree inferred from segment 3. And most importantly, in the segment 3 tree (Fig. 4 right), the coronavirus RaTG13 branches outside the well supported bipartition (0.91) defined by the coronaviruses: bat_RShSTT182, Guangdong 1, Wuhan-Hu-1/2019, BANAL-20-103 and BANAL-20-52, thus supporting the hypothesis that the RBM in RaTG13 was acquired via recombination with a yet unknown coronavirus (Boni et al. 2020).

We further evaluated the phylogenetic dissonance (*D*) between the two trees in Fig. 4 by using GALAX software. Dissonance is a measure of phylogenetic conflict between segments/partitions of data; and is estimated by measuring the average information content in Bayesian posterior tree samples from individual segments minus the information contained in the merged set of Bayesian tree samples from all segments (Lewis et al. 2016). *D* takes values from 0 to 1 (or 0 to 100%) where 0 indicates no phylogenetic conflict between segments. Dissonance between two trees can be further partitioned by clades. This is, it is possible to identify which clades contribute most to dissonance between trees.

In Fig. 4 we show which clades contribute most to dissonance (*D*) between trees. The largest percentage to dissonance (43%) is contributed by the partition that divides the tree between the external group (Rco319) and the rest of the OTUs. This is expected because the two trees are different as a whole. However, the second percentage to dissonance is contributed by the partition containing coronaviruses most closely related to Wuhan-Hu-1/2019, including RaTG13 (69%–43% = 26%), these are depicted with red doted lines connecting the two trees. On the third place, is the contribution to dissonance of the partition that includes the above species plus GuanxiP4L and RSYN04 (56%–43% = 13%), these are depicted with orange lines. This result further reinforces that segment 3 has a different phylogenetic history than segments 2 and 4. The coverage of the dissonance analysis is 0.78 (see supplementary material for a complete description of the statistics associated with the dissonance analysis).

The origin of the RBM by recombination in RaTG13 was further confirmed by analysis with the Recombination Detection Program (RDP) (Martin et al. 2020). This software applies several different methodologies to a set of sequences and calculates an overall consensus score to assess the veracity of detected recombination events. A full exploratory recombination scan identified a recombination event with high confidence (consensus score > 60) between RaTG13 and an unknown coronavirus at positions 1308 to 1514 of the multiple sequence alignment. These coordinates correspond to the RBM and coincides with that detected by GARD (see supplementary material).

The next question is whether the immediate co-descendant to the clade conformed by Wuhan-Hu-1/2019, BANAL-20-52 and BANAL-20-103 is the bat (bat_RShST188) or the pangolin (Guangdong 1) coronavirus. This is important because it would indicate if the RBM from Wuhan-Hu-1/2019 descend from a coronavirus that infects bats or pangolins.

Given that the posterior probability of the node supporting the close relationship of Guangdong 1 to the clade containing Wuhan-Hu-1/2019, BANAL-20-52 and BANAL-20-103 in the tree inferred from segment 3 is low (0.58; Fig. 4, right), one possibility is that the closest coronavirus to the clade containing Wuhan-Hu-1/2019 is bat_RShST188, as shown in the tree inferred from segments 2 and 4 in Fig. 4 (left). In fact, an alternative phylogeny to that shown in Fig. 4 (right) were Guangdong 1 shifts position with bat_RShST188, is not significatively worse than the original tree according to a Kishino-Hasegawa test (*p* value = 0.341) (see supplementary material). Therefore, the hypothesis that the RBM from SARS-CoV-2 evolved from a bat infecting coronavirus cannot be rejected.

**Fig. 5:**
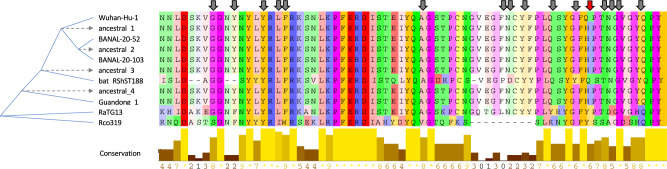
Ancestral sequence reconstruction shows that the RBM of the common ancestor of Wuhan-Hu-1/2019, BANAL-20-52, BANAL-20-103, bat_RShST188 and Guangdong 1 (here named as “ancestral_4”) was identical to the RBM of Wuhan-Hu-1/2019, except for the residue Q498H (red arrow). Amino acids involved in human ACE2 recognition are indicated with arrows

If we use this alternative topology where Guangdong 1 shifts position with bat_RShST188 to reconstruct the ancestral sequences, we find that the RBM of the common ancestor of Wuhan-Hu-1/2019 and Guangdong 1 (the sequence named as “ancestral 4” in Fig. 5) was identical to that of Wuhan-Hu-1/2019, with the exception of residue Q498H. Showing that natural selection did not favor changes in the RBM of these coronaviruses to adapt to new hosts since they last shared a common ancestor. The same result is obtained if the original tree (the one shown in Fig. 4 right) is used for the ancestral sequence reconstruction (see supplementary material).

## Discussion

The analyses provided here shows that the RBM from RaTG13 is not closely related to the RBM from SARS-CoV-2 and was likely acquired by recombination with a yet unknown coronavirus (Boni et al. 2020). Because of that, the RBM from the coronaviruses from Laos (BANAL-20-52 and BANAL-20-103) are the most closely related to the RBM from SARS-CoV-2 (Temmam et al. 2022).

Our results also show that SARS-CoV-2 did not acquire its RBM by recombining with a pangolin infecting coronavirus. Instead, our analyses indicate that the coronaviruses Wuhan-Hu-1/2019, BANAL-20-52 and BANAL-20-103 inherited its RBM most likely from a bat infecting coronavirus. Parsimony favors this interpretation given that bat_RShST188, Wuhan-Hu-1/2019, BANAL-20-52 and BANAL-20-103 are all bat-infecting coronaviruses. If recombination between a bat and pangolin infecting coronaviruses played a role in the evolution of the RBM (or the whole RBD), this may have occurred prior to the divergence of bat_RShST188 and Wuhan-Hu-1/2019.

Our results are in agreement with the interpretation of Temmam et al. (2022) regarding the evolution of the RBM in SARS-CoV-2. Accordingly, natural selection did not incidentally improve the affinity of the RBM for human ACE2 in an intermediate host before spillover (Makarenkov et al. 2021), nor did selection optimize the RBM in humans early after spillover (Andersen et al. 2020). This follows from the fact that the RBM from SARS-CoV-2 is identical to the ancestral sequence it shared with Guangdong 1 with the exception of a single amino acid change Q498H. The conservation of the RBM between coronaviruses that infects pangolins, bats and humans is consistent with recent research showing that SARS-CoV-2 is a generalist virus that is not specifically adapted to humans (Li et al. 2023). However, the origin(s) of other peculiarities of SARS-CoV-2, like the furin-cleavage site, remain to be elucidated. Such features may have evolved by different mechanisms that may have included the passage of the coronavirus in an intermediate host.

## Material and Methods

Gene sequences from the spike protein were retrieved from the GenBank and GISAID databases (https://gisaid.org/; Khare et al. 2021). Author acknowledgments for sequences downloaded from GISAID are provided in supplementary material. The spike protein coding genes were extracted from genome sequences following annotation. When annotation was not available, we identified the spike coding gene by aligning the gene from Wuhan-Hu-1/2019 to the query genome using BLAST (https://blast.ncbi.nlm.nih.gov/Blast.cgi). Codon multiple sequence alignment was performed in MEGA software 11v (Tamura et al. 2021).

Recombination analysis was done with GARD as implemented in http://datamonkey.org/ (Weaver et al. 2018) with the following parameters: normal run mode, universal genetic code, without site-to-site rate variation and 2 rate classes.

Domains in the spike protein follow those defined by Lan et al. (2020) and Xia (2021) and residues in spike protein involved in human ACE2 recognition follow those indicated by Temmam et al. (2022).

Pre-calculated conservation profiles were retrieved from ConSurf Web Server (https://consurf.tau.ac.il/consurf_index.php) for the crystal structure 6M0J chain E (Lan et al. 2020). Protein structure was visualized and colored with Chimera (Pettersen et al. 2004).

We followed the approach by Neupane et al. (2019) to test for the CONCATENATED (*M*_C_) and SEPARATED hypothesis/models (*M*_S_). Accordingly, for a given data set ***y*** (sites in a multiple sequence alignment), their marginal likelihoods *p*(***y***|*M*_C_) and *p*(***y***|*M*_S_) are calculated with the stepping stone algorithm as implemented in MrBayes (Ronquist et al. 2012). Next, the Bayes factor BF is:

$${B}_{CS} = \frac{p(y|{M}_{C})}{p(y|{M}_{S})}$$

These authors suggest the following interpretation of BF:

**Table:** 

log_10_(*B*_CS_)	*B*_CS_	Evidence against *M*_S_
0 to ½	1 to 3.2	Not worth more than a bare mention
½ to 1	3.2 to 10	Substantial
1 to 2	10 to 100	Strong
> 2	> 100	Decisive

Phylogenetic dissonance, *D*, was calculated with GALAX software (https://github.com/plewis/galax). To generate the sample trees required for GALAX, we ran the mcmc algorithm in MrBayes with 1,000,000 generations and a sampling frequency of 500. The model was set to: GTR + G + I. Phylogenetic trees from Fig. 4 were inferred with MrBayes with the same parameters and 25% of burnin was discarded from the sample. For stepping-stone analysis the ss algorithm was set to run 1,000,000 generations and sample each 1000 generation. Example files to run mcmc and ss algorithms in MrBayes are provided in supplementary material.

A full exploratory recombination scan was applied to the multiple sequence alignment with the program RDP (Martin et al. 2020). Methods used within RDP were: RDP, GENECONV, BootScan, MaxChi, Chimera, SiScan and 3Seq. Default parameters were used and sequences were assumed to be linear. We further asked RDP to save a distributed alignment with recombinant regions separated. Based on this distributed alignment we inferred a Maximum-Likelihood tree with MEGA11 (100 bootstrap replicas and GTR + G model of sequence evolution). For clarity, we included in this tree only the recombinant sequence corresponding to the RBM from RaTG13.

Figure 3 was generated with Circos (Krzywinski et al. 2009). Kishino-Hasegawa test was implemented in IQ-TREE (Kishino and Hasegawa 1989; Minh et al. 2020). Ancestral sequence reconstruction (ASR) was performed in MEGA software 11v by Maximum-Likelihood under the Tamura-3 parameter model and including all sites (Tamura et al. 2021). Multiple sequence alignment was visualized with Jalview (Waterhouse et al. 2009).

Supplementary material.

### Supplementary Information

Below is the link to the electronic supplementary material.

Supplementary file1 (DOCX 280 KB)
