# Correction: Untangling the Evolution of the Receptor-Binding Motif of SARS-CoV-2

**DOI:** 10.1007/s00239-024-10183-y

**Published:** 2024-06-25

**Authors:** Luis Delaye, Lizbeth Román-Padilla

**Affiliations:** Departamento de Ingeniería Genética, Cinvestav Unidad Irapuato, Km 9.6 Libramiento Norte Carretera Irapuato-León, C.P. 36824 Irapuato, Gto. Mexico

**Correction to: Journal of Molecular Evolution (2024) 92:329–337** 10.1007/s00239-024-10175-y

In this article the phylogenies was missing in Fig. 4; the corrected Fig. 4 should have appeared as shown below.

**Fig. 4:**
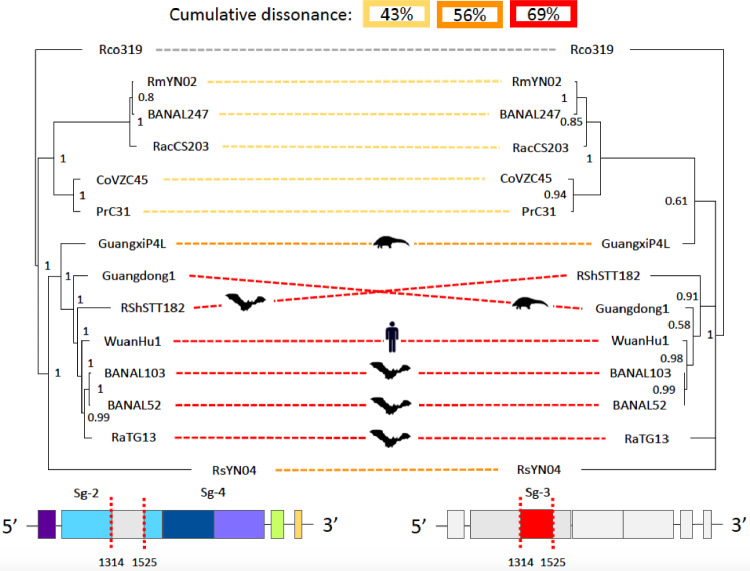
The RBM from RaTG13 has a distinct phylogenetic history than its RBD. Tree on the left is from the concatenation of segments 2 and 4 (containing the RBD); and the tree on the right is from segment 3 (containing the RBM). At the bottom we show segments 2 and 4 (left) and 3 (right) colored by subdomains and motifs. The vertical red lines indicate the recombination breakpoints identified by GARD. At the top of the Figure we show the cumulative dissonance by clades. The hosts are shown for clarity. Note that RaTG13 branches in different clades between the two trees

