# Supplementary material for: Untangling the Evolution of the Receptor-Binding Motif of SARS-CoV-2
Source: J Mol Evol. 2024 May 22;92(3):329–37. doi: 10.1007/s00239-024-10175-y (PMC11168982; doi:10.1007/s00239-024-10175-y)
Supplement: Supplementary file 1 — Supplementary file1 (DOCX 280 KB) [file 239_2024_10175_MOESM1_ESM.docx]

**Supplementary material**

**Untangling the evolution of the Receptor Binding Motif of SARS-CoV-2**

Lizbeth Román-Padilla^1,2^, Luis Delaye^1^*

* Email: luis.delaye@cinvestav.mx

Tel: +52(462) 623-9669

^1^Cinvestav Unidad Irapuato,

^2^Posdoctoral fellow at Cinvestav Unidad Irapuato -Conahcyt

Km 9.6 Libramiento Norte Carretera Irapuato-León C.P. 36824 Irapuato, Gto., México

Index

Page 2: GALAX results from comparing segment 3 with fused segments 2 and 4.

Page 4: Kishino-Hasegawa test with IQ-TREE.

Page 5: Ancestral sequence reconstruction using the topology in Figure 4 (right).

Page 6: Instructions for mcmc and ss algorithms in MrBayes.

Page 7: Results from RDP program.

GALAX results from comparing segment 3 with fused segments 2 and 4

Galax 1.1.0

Options specified:

--treefile:

--listfile: infile

--skip: 200

--rooted: false

--details: true

--outgroup: 5

--outfile: output-galax

--mapto:

--maptorooted: false

Read 1801 trees from tree file segment3.run1.t

Read 1801 trees from tree file segments_2_and_4.run1.t

Order of taxa in split representations:

1 WuhanHu1

2 RaTG13

3 CoVZC45

4 Guangdong1

5 Rco319

6 BANAL52

7 BANAL103

8 BANAL247

9 GuangxiP4L

10 RShSTT182

11 PrC31

12 RacCS203

13 RsYN04

14 RmYN02

Totals

Key to columns:

unique = number of distinct tree topologies

coverage = Larget (2013. Syst. Biol. 62:501–511) estimated posterior coverage

H = entropy of marginal prior tree topology distribution

H* = entropy of marginal posterior tree topology distribution

I = Lindley information (H - H*)

Ipct = I expressed as percent of maximum (100*I/H)

D = dissonance (merged H* - average H*)

Dpct = D expressed as percent of maximum (100*D/(merged H*))

| treefile | unique | coverage | H | H* | I | Ipct | D | Dpct |
| --- | --- | --- | --- | --- | --- | --- | --- | --- |
| segment 3 | 452 | 0.86072 | 26.47975 | 5.43961 | 21.04014 | 79.45747 | --- | --- |
| segs 2 and 4 | 16 | 0.99823 | 26.47975 | 0.66666 | 25.81308 | 97.48236 | --- | --- |
| average | 234 | 0.92948 | 26.47975 | 3.05314 | 23.42661 | 88.46992 | --- | --- |
| merged | 468 | 0.77794 | 26.47975 | 4.16343 | 22.31632 | 84.27693 | 1.11029 | 26.66775 |

Clades sorted by merged info (top 95% shown):

I Ipct cum. Ipct D w clade

24.49571 29.06574 29.06574 0.48533 1.00000 ****-*********

11.19887 13.28818 42.35392 0.07913 1.00000 --*----*--**-*

5.28085 6.26606 48.61998 0.00000 0.17712 *-**-*******-*

4.68627 5.56057 54.18055 0.14493 0.56052 **-*-**-**--*-

4.57130 5.42414 59.60469 0.04568 0.53554 **-*-**-**----

3.92522 4.65752 64.26221 0.28912 0.61105 **-*-**--*----

3.65319 4.33475 68.59696 0.00000 0.49833 **---**--*----

2.96407 3.51706 72.11402 0.00000 0.49972 **---**-------

2.55647 3.03341 75.14743 0.06100 0.92476 -------*---*-*

2.51789 2.98764 78.13507 0.00000 0.30344 --*----**-**-*

2.43567 2.89008 81.02515 0.00000 0.45364 *--*-**--*----

2.40951 2.85904 83.88419 0.00400 0.15186 ****-*******-*

2.06177 2.44642 86.33062 0.00000 0.49695 -*---**-------

1.89757 2.25159 88.58221 0.00002 0.49112 *----**-------

1.75538 2.08287 90.66508 0.00000 0.07440 *-**-*********

1.70907 2.02792 92.69300 0.00000 0.28928 *--*-**-------

0.77172 0.91570 93.60870 0.00000 0.09300 --*----*--****

0.74383 0.88261 94.49130 0.00046 0.06580 **-*-**--*--*-

Clades sorted by D (top 95% shown):

D Dpct cum. Dpct Ipct w clade

0.48533 43.71223 43.71223 24.49571 1.00000 ****-*********

0.28912 26.03965 69.75188 3.92522 0.61105 **-*-**--*----

0.14493 13.05311 82.80499 4.68627 0.56052 **-*-**-**--*-

0.07913 7.12724 89.93223 11.19887 1.00000 --*----*--**-*

Clades sorted by merged clade posterior (w) (only those >= 50% shown):

w Ipct D clade

1.00000 24.49571 0.48533 ****-*********

1.00000 11.19887 0.07913 --*----*--**-*

0.99389 0.00000 0.00000 -----**-------

0.97002 0.00000 0.00000 --*-------*---

0.92476 2.55647 0.06100 -------*---*-*

0.90561 0.00000 0.00000 -------*-----*

0.61105 3.92522 0.28912 **-*-**--*----

0.56052 4.68627 0.14493 **-*-**-**--*-

0.53554 4.57130 0.04568 **-*-**-**----

We show in red and orange the clades shown in Figure 4 with the same colors.

Kishino-Hasegawa test with IQ-TREE

Trees used in file testtrees.treels:

((RaTG13,(((CoVZC45,PrC31),((BANAL247,RmYN02),RacCS203)),GuangxiP4L),RsYN04,(RShSTT182,(Guangdong1,(WuhanHu1,(BANAL52,BANAL103))))),Rco319);

((RaTG13,(((CoVZC45,PrC31),((BANAL247,RmYN02),RacCS203)),GuangxiP4L),RsYN04,(Guangdong1,(RShSTT182,(WuhanHu1,(BANAL52,BANAL103))))),Rco319);

IQ-TREE command:

iqtree2 -s segment3.fasta -z testtrees.treels -n 0 -zb 10000 -redo

IQ-TREE outfile:

USER TREES

----------

See segment3.fasta.trees for trees with branch lengths.

Tree logL deltaL bp-RELL p-KH p-SH c-ELW

------------------------------------------------------------------

1 -1682.028038 0 0.605 + 0.659 + 1 + 0.56 +

2 -1682.328034 0.3 0.396 + 0.341 + 0.341 + 0.44 +

deltaL : logL difference from the maximal logl in the set.

bp-RELL : bootstrap proportion using RELL method (Kishino et al. 1990).

p-KH : p-value of one sided Kishino-Hasegawa test (1989).

p-SH : p-value of Shimodaira-Hasegawa test (2000).

c-ELW : Expected Likelihood Weight (Strimmer & Rambaut 2002).

Plus signs denote the 95% confidence sets.

Minus signs denote significant exclusion.

All tests performed 10000 resamplings using the RELL method.

Ancestral sequence reconstruction using the topology in Figure 4 (right).


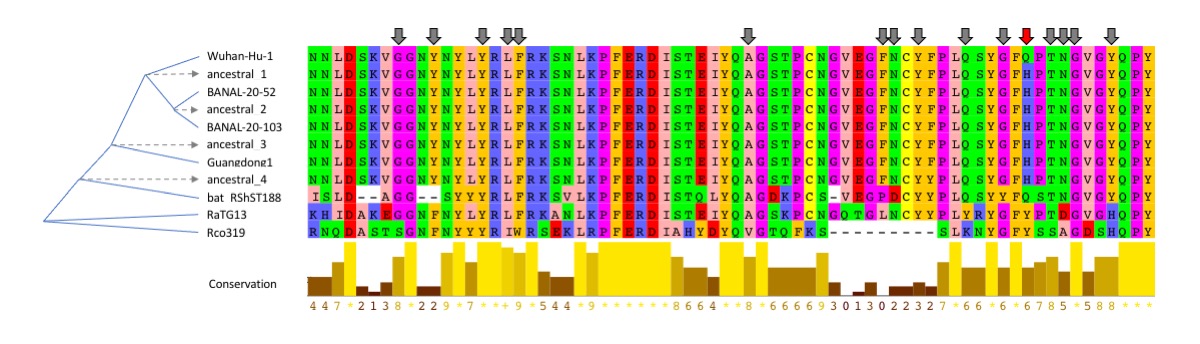


Ancestral sequence reconstruction showing that the RBM of the common ancestor of Wuhan-Hu-1/2019, BANAL-20-52, BANAL-20-103, bat_RShST188 and Guangdong 1 (here named as “ancestral_4”) was identical to the RBM of Wuhan-Hu-1/2019, except for the residue Q498H (red arrow). Amino acids involved in human ACE2 recognition are indicated with arrows. The same result is obtained but with an alternative topology (Figure 5).

Instructions for mcmc algorithm in MrBayes:

exe segment_1_858.nex;

lset nst=6 rates=invgamma ngammacat=4;

prset brlenspr=unconstrained:GammaDir(1.0, 0.01, 1.0, 1.0) shapepr=exp(1.0) statefreqpr=dirichlet(1.0,1.0,1.0,1.0) revmatpr=Dirichlet(1.0,1.0,1.0,1.0,1.0,1.0);

mcmc ngen=1000000 samplefreq=500 printfreq=10000 burninfrac=0.25 nchains=4 nruns=2 file=outfile_1_858.txt;

sump;

sumt;

Instructions for ss algorithm in MrBayes:

exe segment_1_858.nex;

lset nst=6 rates=invgamma ngammacat=4;

prset brlenspr=unconstrained:GammaDir(1.0, 0.01, 1.0, 1.0) shapepr=exp(1.0) statefreqpr=dirichlet(1.0,1.0,1.0,1.0) revmatpr=Dirichlet(1.0,1.0,1.0,1.0,1.0,1.0);

ss ngen=1000000 samplefreq=1000 printfreq=10000 file=outfile_1_858.txt;

**Results from the RDP program**

First, we show the recombination events that were found by a full exploratory recombination scan (Table 1). All these recombination events were detected by all methods used (RDP, GENECONV, BootScan, MaxChi, Chimera, SiScan and 3Seq). All these events were visually reviewed and manually accepted.


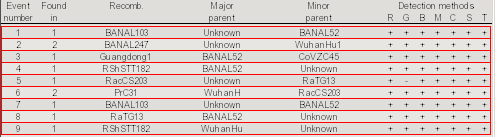


Table 1. Recombination events detected by a full exploratory scan.

Next, we show a diagram of the recombination events (Figure 1). Those recombination events that were accepted are identified with red rectangles. In particular, notice the recombination event in RaTG13 sequence that is from an unknown source, this one correspond to the RBM and correspond to event number 8.


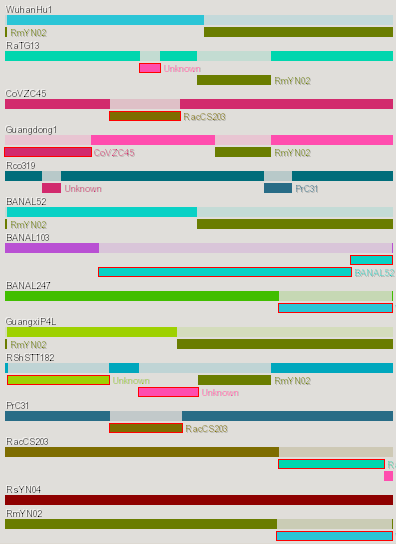


Figure 1. Diagram of recombination events detected with the full exploratory scan. Those in red rectangles are the one that were visually inspected and accepted.

Next, we show for clarity only the results from breakpoint number 8, corresponding to the RBM from RatG13 (Table 2).

| Breakpoint Positions | | | | | |
| --- | --- | --- | --- | --- | --- |
| In Alignment | | In Recombinant Sequence | | Relative to WuhanHu1 | |
| Begin | End | Begin | End | Begin | End |
|  |  |  |  |  |  |
| 1338 | 1544 | 1308 | 1514 | 1308 | 1514 |

Table 2.

| Sequences involved in recombination | | |
| --- | --- | --- |
|  | | |
| Recombinant Sequence(s) | Minor Parental Sequence(s) | Major Parental Sequence(s) |
|  |  |  |
| RaTG13 | Unknown (Guangdong1) | BANAL52, WuhanHu1 |

Table2. Continuation.

| Detection Methods and p-value | | | | | | |
| --- | --- | --- | --- | --- | --- | --- |
|  | | | | | | |
| RDP | GENECONV | Bootscan | Maxchi | Chimaera | SiSscan | 3Seq |
|  |  |  |  |  |  |  |
| 2.82E-09 | 0.00493582 | 1.32E-07 | 5.44E-07 | 0.00320936 | 8.13E-07 | 3.01E-10 |

Table2. Continuation.

Next, we show the overall consensus score of recombinant segment 8 (Figure 2).


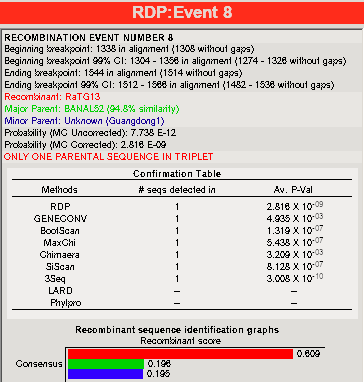


Figure 2. Overall consensus score of the recombination event number 8 corresponding to the RBM from RatG13. A value over 0.6 in the red bar is considered highly significative.


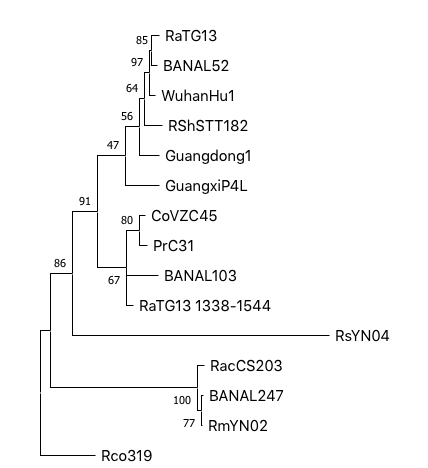


Figure 3. Phylogenetic ML tree of the spike protein where the recombinant segment 8 from RaTG13 corresponding to the RBM (positions 1338 – 1544 in the multiple alignment) has been analyzed separately from the rest of the RaTG13 sequence. The numbers in the nodes correspond to 100 bootstrap replicas.

Finally, we show a phylogenetic tree including non-recombinant segments, plus the recombinant segment 8 corresponding to the RBM from RaTG13 (1338 -1544) (Figure 3). Notice that this recombinant segment branches to a different clade than WuhanHu1 (SARS-CoV-2).
